# *Bacillus subtilis* encodes three *N*-acetylcysteine deacetylase enzymes that can catalyze the final step in *S*-(2-succino)cysteine breakdown

**DOI:** 10.1016/j.jbc.2025.110954

**Published:** 2025-11-17

**Authors:** Anthony J. Zmuda, Andrew J. Toensing, Katie B. Wissbroecker, Thomas D. Niehaus

**Affiliations:** The Department of Plant and Microbial Biology, University of Minnesota, St Paul, Minnesota, USA

**Keywords:** acetylation, bacterial metabolism, post-translational modification (PTM), enzyme catalysis, biodegradation, *Bacillus*

## Abstract

Succination occurs when the TCA cycle intermediate fumarate reacts with cellular thiols, such as cysteine, yielding the damaged metabolite *S-*(2-succino)cysteine (2SC). Increased fumarate levels result in global succination of thiol-containing macromolecules and metabolites, which has been implicated in many human diseases. 2SC is a chemically stable molecule; however, enzymatic breakdown pathways have been identified in prokaryotes that involve *N*-acetylation of 2SC followed by a breakdown step that results in the release of the succino-moiety and *N*-acetylcysteine (NAC). NAC must be metabolized to cysteine to be assimilated, but enzymes catalyzing NAC deacetylation had hitherto not been thoroughly characterized. Here, we describe three enzymes in *Bacillus subtilis*, ScmP, YhaA, and YtnL, that all possess high NAC deacetylase activity *in vitro*. All three enzymes are metal-dependent hydrolases that are most active with cobalt and show remarkable specificity to NAC compared to structurally related acetylated small molecules. Growth assays demonstrated that these genes are functionally redundant in *B*. *subtilis*, and growth on NAC is only severely compromised when all three genes are knocked out of the genome. Together, our biochemical and genetic studies complete the functional characterization of the three-step 2SC degradation pathway in *B*. *subtilis*.

Cysteine residues of peptides and low-molecular-weight thiols (LMWTs) are common targets of alkylation *in vivo* (1, 2, 3, 4). Cysteine, being a nucleophile, reacts spontaneously with electrophilic alkylating agents to form stable thioether bonds, effectively capping the cysteine residue and preventing its participation in other reactions (5, 6). The TCA cycle intermediate fumarate is a prominent physiological alkylating agent of cysteine in a reaction termed succination (1, 2). The succination reaction may involve protonated hydrogen fumarate, which would enhance the reactivity of fumarate to cysteine (7). The thioether product of succination is known as *S-*(2-succino)cysteine (2SC), and this modification is a common and universal post-translational modification (8). Levels of endogenously succinated proteins are directly proportional to fumarate levels (1). In humans, factors such as diabetes (9, 10, 11), obesity (12), and fumarate hydratase deficiency (13, 14) cause fumarate levels to increase, resulting in an increase in global protein succination. Buildup of 2SC is deleterious, as succination inactivates enzymes involved in metabolism, cell structure, and cell cycle (12, 15, 16, 17, 18, 19). Thus, succination likely plays a major role in metabolic aging and the progression of certain diseases.

A pathway to breakdown 2SC and recover the damaged cysteine was first identified in *Bacillus subtilis* using a high-throughput screen (20). The pathway is encoded by the *S-(2-succino)cysteine metabolism* (*scm*) gene cluster (formerly *yxe* gene cluster) and consists of three enzymatic steps (Fig. 1*A*). The breakdown pathway is initiated by the acetyltransferase ScmL, which acetylates 2SC to form *N*-acetyl-2SC (2SNAC) (20). The FMN-dependent monooxygenase ScmK hydroxylates 2SNAC, resulting in the formation of oxaloacetate and N-acetylcysteine (NAC) (20, 21, 22). In the final step, NAC is predicted to be deacetylated by ScmP to release free cysteine (20). While this gene cluster occurs in taxonomically diverse bacteria, the gene encoding the key disassembly step, *scmK*, is often replaced with another gene, *S*-(2-succino)cysteine lyase (2SL), which encodes a lyase enzyme also capable of 2SNAC disassembly *via* a distinct enzymatic reaction (23).

**Figure 1:**
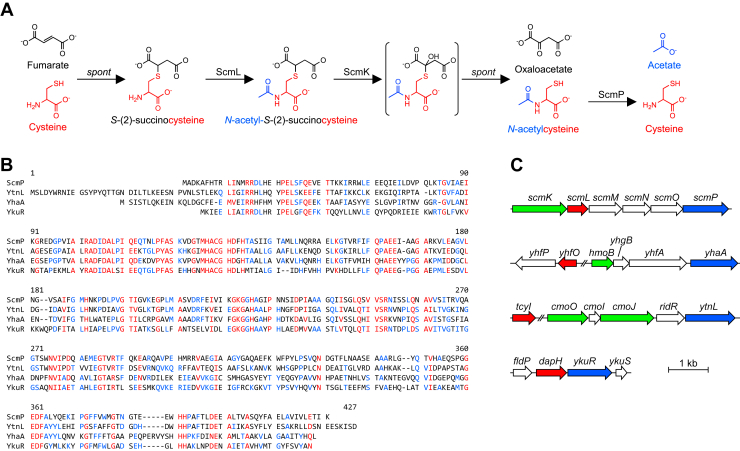
***B*. *subtilis* has multiple gene clusters that putatively function in the breakdown of *S*-alkylcysteine compounds**. *A*, characterized *B*. *subtilis* breakdown pathway for *S*-(2)-succinocysteine *via* enzymes encoded by the *scm* gene cluster. *B*, amino acid alignment of four putative *B*. *subtilis N*-acetylcysteine deacetylases: ScmP, YtnL, YhaA, and YkuR. *Red*, high consensus; *blue*, low consensus; *black*, no consensus. *C*, loci around four putative *N*-acetylcysteine deacetylase genes. Deacetylase genes in the genome (*blue*) commonly cluster with acetyltransferase genes (*red*) and monooxygenase genes (*green*).

In addition to the *scm* operon, *B*. *subtilis* encodes two other biosynthetic gene clusters (*yha* and *ytn*) predicted to catabolize *S*-modified cysteines (Fig. 1*B*). There is evidence that they act on different *S*-modified cysteine substrates such as *S*-methylcysteine, *S*-benzylcysteine, and *S*-hydroxylcysteine (22, 24). Both clusters encode predicted acetyltransferases, monooxygenases, and deacetylases, similar to the *scm* operon (Fig. 1*C*), and *S*-alkylcysteine breakdown is predicted to mirror that of 2SC using a strategy of acetylation, disassembly, and deacetylation. In all cases described to date, acetylation is crucial for breakdown; thus, NAC is the common predicted reaction product in the breakdown of *S*-alkylcysteine adducts catalyzed by these gene clusters.

All steps of the 2SC breakdown pathway are well-characterized except NAC deacetylation. Here, we study NAC deacetylation in *B*. *subtilis* biochemically and genetically. We show that *B*. *subtilis* has three redundant NAC deacetylase genes that can all catalyze the final step in breakdown pathways of 2SC and other alkylated cysteine compounds.

## Results

### Identification of putative NAC deacetylase enzymes in *B*. *subtilis*

Based on sequence similarity to ScmP (BSU39470; UniProt ID: P54955), we identified three additional NAC deacetylase candidate genes in the *B*. *subtilis* genome: *yhaA* (BSU10070; UniProt ID: O07598), *ytnL* (BSU29290; UniProt ID: O34980), and *ykuR* (BSU14190; UniProt ID: O34916). The proteins encoded by these genes all belong to the M20 peptidase protein family, and they share between 35% and 50% sequence identity with one another (Fig. 1*B*).

The *scmP*, *yhaA*, and *ytnL* genes are encoded in biosynthetic gene clusters predicted to be involved in recovering cysteine after it is damaged by alkylating agents (Fig. 1*C*). In addition to the predicted *N*-acetylcysteine deacetylase gene, each gene cluster contains an acetyltransferase gene and a monooxygenase gene (Fig. 1*C*) that likely participate in the breakdown of different *S*-alkylcysteine compounds *via* multistep pathways involving acetylation, breakdown, and deacetylation (22, 24). The *scm* gene cluster is involved in the breakdown of 2SC caused by fumarate damage, but it’s unclear what specific *S*-alkylcysteine compound(s) the *yha* and *ytn* gene clusters target. Regardless of the specific *S*-alkylcysteine compound, after acetylation and cleavage of the adduct, *N*-acetylcysteine is the expected product, and a deacetylation step is required to recover cysteine (Fig. 1*A*). Thus, while these gene clusters may function to repair various cysteine damage products, the deacetylases encoded in each cluster are likely redundant, so we considered *scmP*, *yhaA*, and *ytnL* genes strong *N*-acetylcysteine deacetylase candidates.

The *ykuR* gene is essential in *B*. *subtilis* and encodes an enzyme with *N*-acetyldiaminopimelate deacetylase activity that is an essential step in peptidoglycan synthesis (25, 26, 27, 28). However, we still considered it a viable *N*-acetylcysteine deacetylase candidate due to sequence conservation with ScmP and potential for dual *N*-acetyldiaminopimelate and *N*-acetylcysteine deacetylation activities.

### ScmP, YhaA, and YtnL catalyze the release of cysteine from *N*-acetylcysteine

**Figure 2:**
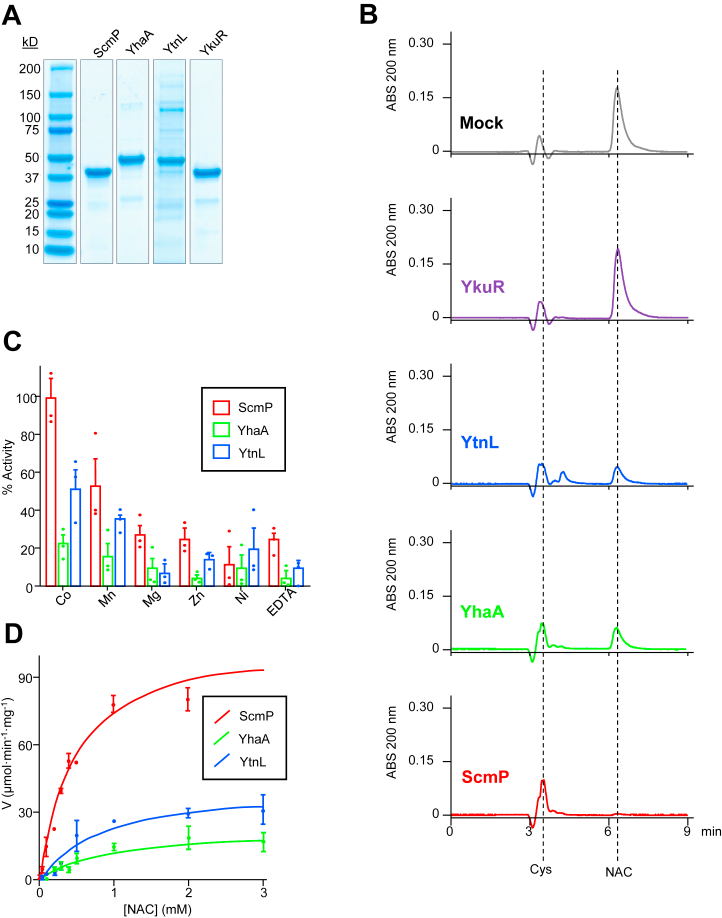
**ScmP, YhaA, and YtnL all have NAC deacetylase activity**. *A*, SDS-PAGE analysis of enriched recombinant *B*. *subtilis* enzymes as indicated. *B*, HPLC analysis of NAC deacetylase activity of *B*. *subtilis* enzyme candidates. Assays (50 μl) contained 50 mM potassium phosphate (pH 7.8), 5 mM MgCl_2_, 4 mM NAC, and either 1.0 μg of purified ScmP, YhaA, YtnL, or YkuR or an equal volume of enzyme storage buffer (mock). Reactions were incubated for 10 min at 37 °C and stopped with the addition of 2 μl 1M HCl. 25 μl of each reaction was analyzed *via* RP-HPLC with detection at 200 nm. *C*, effect of metal on NAC deacetylase activity. Assays (50 μl) contained 50 mM potassium phosphate (pH 7.8), 2 mM NAC, 5 mM of metal salt (CoCl_2_, MnCl_2_, MgCl_2_, ZnCl_2_, NiCl_2_) or 10 mM EDTA, and 0.1 μg of purified ScmP, YhaA, or YtnL. Reactions were incubated for 4 min at 37 °C and stopped with the addition of 2 μl 1M HCl. HPLC analysis was performed as described above. Data represents mean and S.E.M. of three independent experiments. *D*, Michaelis-Menten kinetics of *B*. *subtilis* NAC deacetylases. Assays were run and analyzed as in (*C*), but with 5 mM CoCl_2_ and varying concentrations of NAC. Data points represent mean and S.E.M. of three independent experiments.

Based on sequence homology and/or genomic context (Fig. 1, *B* and *C*), we predicted that *B*. *subtilis* may encode up to four enzymes with *N*-acetylcysteine deacetylase activity. To produce proteins for use in enzyme assays, we cloned each gene's coding sequence into the pET28b vector, expressed recombinant enzymes containing *N*-terminal hexahistidine tags in *E*. *coli*, and enriched enzymes *via* nickel affinity chromatography. All four enzymes were shown to be highly enriched by SDS-PAGE analysis (Fig. 2*A*).

To initially test for NAC deacetylase activity, we developed assays in which both the expected substrate NAC and product cysteine could be chromatographically separated and quantified using reverse-phase high-performance liquid chromatography (RP-HPLC) with absorbance detected at 200 nm. Under these chromatographic conditions, cysteine and NAC eluted at 3.7 min and 6.0 min, respectively, and the molar absorbance of NAC at 200 nm was 3.5-fold higher than that of cysteine (Supplemental Fig. S1). Assays contained 50 mM sodium phosphate, pH 7.8, 5 mM MgCl_2_, 4 mM NAC, without or with 1.0 μg recombinant enzyme, and were incubated for 10 min at 37 °C before being stopped with the addition of 2 μl 1M HCl. Addition of ScmP caused the near-total disappearance of NAC, and the appearance of a stoichiometric amount of cysteine (Fig. 2*B*). Addition of YhaA or YtnL caused NAC to decrease by 69% or 76%, respectively, with the formation of a stoichiometric amount of cysteine (Fig. 2*B*). No disappearance of NAC or formation of cysteine was observed upon addition of YkuR. These results indicate that ScmP, YhaA, and YtnL proteins have NAC deacetylase activity, with ScmP apparently having the highest activity. YkuR lacked measurable NAC deacetylase activity with our assay conditions.

Cysteine is difficult to detect by HPLC because it does not strongly absorb ultraviolet light and because it elutes close to the injection front. We also analyzed the ScmP reaction described above *via* single quadrupole liquid chromatography-mass spectrometry (LC-MS) to verify that we were observing the production of cysteine in our assays. When analyzed by LC-MS, NAC and cysteine produced spectra with readily detectable [M + H] molecular ions. In the mock control reaction, we detected NAC and a small amount of cysteine, that was likely due to fragmentation and loss of the acetyl-group during ionization in the LC-MS electrospray chamber. The addition of ScmP resulted in a total consumption of NAC from the reaction, and an increase in peak size for cysteine (Supplemental Fig. S3). These results verify that ScmP deacetylates NAC to cysteine.

### ScmP, YhaA, and YtnL are metal-dependent deacetylases

Initially, we supplemented our assays with magnesium, since we expected these hydrolases would require a divalent cation. Once we determined which enzymes were active against NAC, we tested various metal cofactors to determine their effect on enzyme activity. All three enzymes showed maximum activity when supplemented with cobalt (Fig. 2*C*). Although not to the magnitude of cobalt, manganese significantly stimulated activity relative to magnesium and an EDTA control (Fig. 2*C*). Magnesium, zinc, and nickel did not significantly stimulate enzyme activity relative to an EDTA control (Fig. 2*C*). The highest enzyme activity was observed at pH 7.8 (Supplementary Fig. S4). Accordingly, we performed a rigorous kinetic analysis of each enzyme at pH 7.8 in the presence of 5 mM CoCl_2_ and generated Michaelis-Menten curves (Fig. 2*D*). We found ScmP to be the most catalytically efficient enzyme, with a *K*_*cat*_*·K*_*m*_^*-1*^ of 1.7 × 10^6^ M^-1^ s^-1^ (Table 1). YtnL and YhaA had operable NAC deacetylase activity, although they were less efficient than ScmP, at 4.0 × 10^5^ M^-1^ s^-1^ and 2.1 × 10^5^ M^-1^ s^-1^, respectively (Table 1).

**Table 1:** Kinetic Parameters of putative *B*. *subtilis* NAC deacetylases

Enzyme	Kinetic parameter			
	v_max_ (μmol·min^-1^ mg^-1^)	K_m_ (μM)	K_cat_ (s^-1^)	K_cat_/K_m_ (M^-1^ s^-1^)
ScmP	112.4 ± 6.7	494.5 ± 67.4	82.5 ± 9.2	1.7E6 ± 1.4E5
YhaA	24.7 ± 4.0	895.8 ± 338.1	18.8 ± 1.3	2.1E4 ± 3.7E3
YtnL	43.0 ± 6.7	855.0 ± 339.2	34.2 ± 3.8	4.0E4 ± 1.1E4
YkuR	n.d.			

### ScmP is highly specific for *N*-acetylcysteine

**Figure 3:**
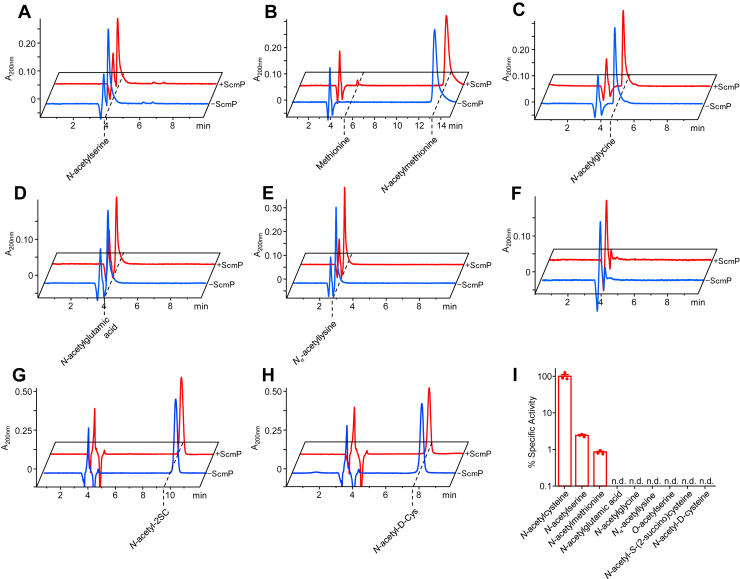
**ScmP has minor activity against *N*-acetylmethionine and *N*-acetylserine**. Assays (50 μl) consisted of 50 mM potassium phosphate (pH 7.8), 2 mM CoCl_2_, and 5 mM of either *N*-acetylserine (*A*), *N*-acetylmethionine (*B*), *N*-acetylglutamic acid (*C*), *N*-acetylglycine (*D*), *N*_⍺_-acetyllysine (*E*), *O*-acetylserine (*F*), *N*-acetyl-*S*-(2-succino)cysteine (*G*), or *N*-acetyl-D-cysteine (*H*). Assays were started with the addition of either 1.0 μg ScmP (*red traces*) or enzyme storage buffer (*blue traces*), incubated for 15 min at 37 °C, then stopped with the addition of 2 μl 1M HCl. 10 μl of each reaction was analyzed by HPLC with detection at 200 nm. *I*, comparison of percent activities of ScmP with various acetylated small molecules. Data is plotted on a logarithmic scale.

To test whether the activity of ScmP is specific toward NAC or if it can act promiscuously on other substrates, we performed our RP-HPLC assays with a variety of acetylated small-molecule substrates. We detected minor activity against *N*-acetylserine and *N*-acetylmethionine (Fig. 3, *A* and *B*), and no activity against *N*-acetylglutamic acid, *N*-acetylglycine, *N*_α_-acetyllysine, or *O*-acetylserine (Fig. 3, *C* and *D*, *E*, *F*). When compared against the specific activity for NAC, we found ScmP to possess 2.8% activity against *N*-acetylserine and 1.0% activity against *N*-acetylmethionine (Fig. 3*I*, Table 2), suggesting that ScmP is highly specific for NAC.

**Table 2:** Percent Specific Activity of ScmP against acetyl-amino acids

Substrate	% activity
*N*-acetylcysteine	100 ± 17.3
*N*-acetylserine	2.83 ± 0.20
*N-*acetylmethionine	0.96 ± 0.09
*N*-acetylglutamic acid	n.d.
*N*-acetylglycine	n.d.
*N*_⍺_-acetyllysine	n.d.
*O*-acetylserine	n.d.
*N*-acetyl-D-cysteine	n.d.
*N*-acetyl-*S*-(2-succino)cysteine	n.d.

We also tested ScmP against *N*-acetyl-*S*-(2-succino)cysteine (2SNAC), the precursor to NAC in the 2SC breakdown pathway (Fig. 1*A*). We found no detectable activity against 2SNAC (Fig. 3*G*), indicating that the succino-moiety must first be cleaved before deacetylation can occur. We also found no detectable activity against *N*-acetyl-D-cysteine (Fig. 3*H*), indicating a stereoselective active site. For all alternative substrates, we validated our results *via* LC-MS by comparing known standards of substrates and products (Supplementary Fig. S2) to reaction end products in mock and experimental samples (Supplementary Fig. S3). These results confirmed minor activity against *N*-acetylserine and *N*-acetylmethionine, and no detectable activity against the other substrates tested.

### ScmP, YhaA, and YtnL are redundant *in vivo*

To assess whether the *B*. *subtilis* NAC deacetylase genes are functional *in vivo*, we obtained single gene knockouts, generated multiple knockout strains of *B*. *subtilis*, and assessed their growth on various sulfur sources. *B*. *subtilis* can use cysteine as a sole sulfur source, so we hypothesized that NAC could be used if it was deacetylated to cysteine. We obtained BKE and/or BKK *B*. *subtilis* mutants (29) for *scmP*, *yhaA*, and *ytnL* in which each gene is replaced with either an erythromycin or kanamycin antibiotic resistance cassette (Supplemental Table 2). Because *ykuR* is an essential gene in *B*. *subtilis*, we were unable to obtain a knockout. To generate a double mutant, we transformed a *ΔscmP*:*erm*^*R*^ strain with genomic DNA from a *ΔyhaA*:*kan*^*R*^ strain. We then removed the antibiotic resistance cassettes from the genome by introduction of the pDR244 plasmid encoding a *cre/lox* recombinase. To generate a triple mutant, we took our double mutant and transformed it with genomic DNA from a *ΔytnL*:*erm*^*R*^ strain. We once again flipped out the resistance cassette, yielding a strain with clean deletions of all three genes.

**Figure 4:**
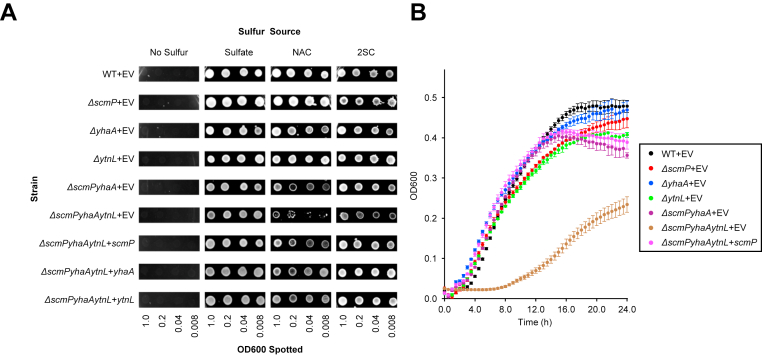
***scmP*, *yhaA*, and *ytnL* encode redundant NAC deacetylases in *B*. *subtilis***. The pHCMC04 empty vector (EV) was transformed into the following *B*. *subtilis* strains: 168 (WT), *ΔscmP*, *ΔyhaA*, *ΔytnL*, *ΔscmPyhaA*, and *ΔscmPyhaAytnL*. The *ΔscmPyhaAytnL* strain was also transformed with the pHCMC04 vector harboring the coding sequencing for *scmP*, *yhaA*, and *ytnL*. Positive transformants were grown overnight at 37 °C in ED media supplemented with 2.5 mM Na_2_SO_4_, washed twice in ED media lacking sulfur, and serially diluted to absorbances of 1.0, 0.2, 0.04, and 0.008. *A*, Five microliters of each dilution were spotted onto ED minimal medium plates with 0.2% (w/v) xylose and either 2 mM Na_2_SO_4_, 0.25 mM NAC, 0.25 mM 2SC, or with no added sulfur. Plates were incubated at 37 °C for 48 h prior to imaging. *B*, 15 microliters of washed cells at an absorbance of 1.0 were added to 285 μl of ED media supplemented with 20 mM NAC in a 48-well plate. Cultures were incubated in a BioTek Epoch 2 Microplate Spectrophotometer at 37 °C for 24 h with continuous shaking, and OD_600_ measurements were taken every 30 min. Data represent the mean and S.E.M. of three independent replicates.

We assessed the growth of wild type, single knockout (*ΔscmP*, *ΔyhaA*, *ΔytnL*), double knockout (*ΔscmPyhaA*), and triple knockout (*ΔscmPyhaAytnL*) *B*. *subtilis* strains on various sulfur sources. On solid medium, all strains grew well on Na_2_SO_4_ (Fig. 4*A*), indicating that loss of these genes does not significantly affect growth on standard medium. However, when given NAC or its precursor 2SC as a sole sulfur source, growth of the triple knockout strain was noticeably compromised, but not that of the single or double knockout strains (Fig. 4*A*). Growth of the triple knockout could be restored, however, with complementation of any of three deacetylase genes (Fig. 4*A*). When grown in liquid medium with NAC as the sole sulfur source, only the triple mutant had severely compromised growth, and growth rate increased with complementation of *scmP* (Fig. 4*B*). These results indicate that *scmP*, *yhaA*, and *ytnL* all function redundantly as NAC deacetylases *in vivo*.

## Discussion

Our results show that *B*. *subtilis* encodes three metal-dependent hydrolases (ScmP, YhaA, and YtnL) that deacetylate NAC to cysteine *in vitro* (Fig. 2*B*). The activity of all three enzymes is highest when incubated with cobalt, but manganese also significantly stimulated activity (Fig. 2*C*). The turnover number (*K*_cat_) of ScmP is 4.4-fold and 2.4-fold higher than that of YhaA and YtnL, respectively (Table 1). ScmP also has a higher affinity for NAC with a *K*_*M*_ nearly half that of YhaA and YtnL (Table 1). Thus, the catalytic efficiency (*K*_cat_·*K*_*M*_^-1^) of ScmP is 8.1-fold and 4.3-fold greater than that of YhaA and YtnL (Table 1). The activity of ScmP is highly specific for NAC over structurally similar acetylated small metabolites. Minor activities were detected against *N*-acetylserine and *N*-acetylmethionine, but the specific activities are around two orders of magnitude lower than that for NAC (Fig. 3). No activity was detected against six other acetylated small molecules, including *N*-acetyl-*D*-cysteine, indicating that ScmP is highly specific and stereoselective for the *L* form of NAC (Fig. 3). All three genes appear to have redundant functions *in vivo*. A triple knockout strain (*ΔscmPyhaAytnL*) was severely limited in its ability to use NAC as a nitrogen source, but not a double knockout (*ΔscmPyhaA*) or single knockout strain (Fig. 4, *A* and *B*). Growth of the triple knockout on NAC was restored when complemented with any of the three genes (Fig. 4*A*). The minor growth rate of the triple knockout (Fig. 4, *A* and *B*) suggests that another enzyme may be slowly deaceylating NAC. Although we did not detect activity against NAC in our *in vitro* assay with YkuR (Fig. 2*B*), this enzyme shares sequence homology to ScmP, YhaA, and YtnL and may have enough activity against NAC to allow for slight growth.

Biochemical and genetic characterization of ScmP function completes the characterization of the entire 2SC breakdown pathway encoded by the *scm* gene cluster in *B*. *subtilis*. The first step in the breakdown pathway is *N*-acetylation of 2SC catalyzed by ScmL. Recombinant ScmL is highly efficient at acetylating 2SC (20), and *B*. *subtilis* Δ*scmL* knockouts are not able to grow on 2SC as a sulfur source (20). These results indicate that 2SC acetylation is a critical step in the breakdown pathway and that this reaction is primarily catalyzed by ScmL in *B*. *subtilis*. The next step in the breakdown pathway is hydroxylation of 2SNAC catalyzed by ScmK, which results in the formation of NAC and oxaloacetate. ScmK is an FMN-dependent monooxygenase that is specific for 2SNAC; no activity was detected against the unacetylated substrate 2SC in assays with recombinant enzyme (21, 22). *B*. *subtilis* Δ*scmK* knockouts are not able to grow on 2SC as a sulfur source (20) indicating that 2SNAC breakdown is primarily catalyzed by ScmK in *B*. *subtilis*. We show here that the final step in the breakdown pathway can be catalyzed by ScmP, which is an efficient NAC deacetylase. Unlike the first two steps, the final step in the breakdown pathway can be catalyzed by three redundant enzymes, ScmP, YhaA, and YtnL in *B*. *subtilis*.

The pathway of 2SC catabolism involving three distinct acetylation, breakdown, and deacetylation steps appears to be highly conserved. The *scm* gene cluster is found in several taxonomically diverse bacterial genomes (20). These gene clusters typically contain a 2SC acetyltransferase *scmL* homolog and an NAC deacetylase *scmP* homolog. However, the gene encoding the 2SNAC breakdown enzyme ScmK is often replaced with another gene, 2SL (23). 2SL enzymes also catalyze the breakdown of 2SNAC, but *via* a lyase reaction that releases fumarate and NAC. This reaction is completely distinct from the FMN-dependent 2SNAC monooxygenase reaction catalyzed by ScmK, which shares no sequence homology to 2SL (23). Intriguingly, while the critical breakdown step of 2SC catabolism can be catalyzed by various unique enzymes, the overall strategy of acetylation, breakdown, and deacetylation still occurs. This general strategy is apparently used in the catabolism of other alkylated cysteine damage products. The *yha* and *ytn* gene clusters have been implicated in the breakdown of alkylated-cysteine compounds such as *S*-methylcysteine and *S*-benzylcysteine (22, 24) through pathways involving acetylation, breakdown, and deacetylation. Showing that ScmP, YhaA and YtnL are *bona fide* NAC deacetylases supports a role in recovering damaged cysteine for their respective gene clusters since NAC is the common penultimate intermediate in all three breakdown pathways.

Apart from being the penultimate intermediate in the breakdown of S-alkylcysteine compounds, NAC is a bioactive compound that has been studied in several contexts. NAC has historically been used in the treatment of respiratory and pulmonary illnesses (30, 31), paracetamol overdose (32), and HIV infection (33). Moreover, NAC appears to possess anticarcinogenic and antimutagenic properties (34, 35). NAC is an antioxidant that functions as a reactive oxygen species scavenger because of its ability to react with electrophiles (36). NAC can also serve as a precursor for the synthesis of glutathione (36), which itself is a prevalent antioxidant held in high intracellular concentrations (37). NAC also has antibacterial properties. In *B*. *subtilis*, NAC concentrations above 3.0 mM begin to inhibit growth, and growth is completely inhibited at 12 mM (38). In other bacteria, NAC exposure has been shown to increase resistance to antibiotics such as doxycycline (39). Characterization of enzymes involved in NAC metabolism provides a new avenue to study the biological effects of NAC exposure.

The dependence on acetylation as a first step in *S*-alkylcysteine breakdown may reflect metabolic advantages that drove the evolution of breakdown pathways. The acetylation-breakdown-deacetylation strategy is analogous to an organic chemist protecting a reactive group during chemical synthesis. A good example of acetylation being used as a protecting/deprotecting strategy in a metabolic pathway comes from arginine synthesis. Glutamate is first *N*-acetylated before being converted to ornithine and deacetylated (40). If the glutamate semialdehyde intermediate wasn’t blocked with an acetyl group, it would rapidly and spontaneously cyclize to 1-pyrroline-5-carboxylate (40, 41). In this case, blocking is required to effectively synthesize ornithine instead of forming a side-product unrelated to arginine synthesis. Acetylation is also used to remove toxic or unwanted compounds, such as the case of the *lac* operon (*lacZYA)*. The lactose permease LacY pumps lactose into cells to potentially toxic levels. LacA is a low-affinity acetyltransferase that acetylates excess lactose, which is then selectively excreted (42). LacA thus acts as a “pressure relief valve” preventing lactose toxicity (43). Acetylation of antibiotics is a common strategy for microbes to evolve resistance, likely because acetyltransferases are diverse enzymes that consist of several protein families acting on a wide range of substrates and can quickly evolve to act on bioactive compounds (44, 45, 46). A similar scenario seems likely for 2SC, which inhibits the growth of *B*. *subtilis* lacking the *scm* gene cluster, but not the *N*-acetyl-derivative 2SNAC, indicating that 2SC is a bioactive compound (20). Thus, the evolution of ScmP to acetylate and neutralize this universal cysteine damage product would provide a selective advantage. Enzymes involved in recovering cysteine from 2SNAC could then evolve.

The breakdown pathways encoded by the *scm*, *yha* and *ytn* gene clusters can be thought of as metabolite repair pathways involved in recovering cysteine that has been damaged through conjugation with electrophilic compounds (19). However, it is not free cysteine but LMWTs such as glutathione (GSH) or cysteine residues of proteins that are more likely targets of succination under physiological conditions because of their abundance and because their sulfhydryl groups can be more acidic and reactive than those of free cysteine (22). At least in the case of 2SC breakdown, the enzymes are only active on free 2SC and not succinated proteins or succinated GSH (23), indicating that damage repair involves hydrolysis of larger biomolecules into free 2SC. 2SC is a universal damaged metabolite for which the *scm* gene cluster evolved to deal with (20, 23). The *ytn* and *yha* gene clusters can breakdown some cysteine adducts such as *S*-methylcysteine, *S*-benzylcysteine, and *S*-hydroxylcysteine that are not expected to be universal damage products (22, 24). These gene clusters may have wide substrate specificities providing general breakdown of various xenobiotic-cysteine adducts, or they may have yet-to-be-discovered substrates (47). Overall, these observations highlight the diversity of repair mechanisms that cells have evolved to recycle damaged cysteine-containing biomolecules.

## Experimental procedures

### Bioinformatics

Amino acid sequences of *B*. *subtilis* 168 ScmP, YhaA, YtnL, and YkuR were obtained from the UniProt database (http://www.uniprot.org/). Sequences were aligned using a multiple sequence alignment through MultAlin (http://multalin.toulouse.inra.fr/multalin/).

### Chemicals

*N*-acetylglutamic acid, glutamic acid, *O*-acetylserine, serine hydrochloride, and glycine were purchased from Thermo-Fisher Scientific. Methionine and cysteine hydrochloride were purchased from Research Products International. *N*-acetylcysteine, *N*-acetylmethionine, *N*-acetylglycine, *N*-acetylserine, and *N*_α_-acetyllysine were purchased from Sigma-Aldrich. Lysine was purchased from AstaTech. *S*-(2-succino)cysteine was purchased from SynInnova. *N*-acetyl-D-cysteine was purchased from AmBeed. All other chemicals were purchased from Sigma-Aldrich. *N*-acetyl-*S*-(2-succino)cysteine was synthesized as described previously (23).

### Constructs for protein expression and complementation assays

The coding sequences for *B*. *subtilis* ScmP (UniProt ID: P54955), YhaA (UniProt ID: OO7598), YtnL (UniProt ID: O34980), and YkuR (UniProt ID: O34916) were amplified from *Bacillus subtlis* genomic DNA with Phusion polymerase (New England Biolabs) using primers listed in Supplementary Table 1. Amplicons were digested with restriction enzymes NheI and XhoI (*scmP* and *ytnL*) or NheI and HindIII (*yhaA* and *ykuR*) and ligated into the matching sites of pET28b to facilitate expression with an N-terminal hexahistidine tag.

For complementation assays, coding sequences were amplified as described above, digested with SpeI and BamHI (*scmP* and *yhaA*) or SpeI and XmaI (*ytnL*), and ligated into the matching sites of pHCMC04. Constructs were verified by Sanger sequencing.

### Construction of mutant *B*. *subtilis* strains

The following strains of *B*. *subtilis* were obtained from the *B*. *subtilis* BKE and BKK libraries (29): BKE39470 (*ΔscmP*:*erm*^*R*^), BKK10070 (*ΔyhaA*:*kan*^*R*^), and BKE29290 (*ΔytnL*:*erm*^*R*^). Generation of mutants was performed as described previously (29). Briefly, from each strain, genomic DNA was isolated, and gDNA from BKK10070 was transformed into BKE39470 and recovered in media containing 10 μg⋅mL^-1^ kanamycin. Successful recombination of *yhaA* with the *kan*^*R*^ cassette in the genome was verified by PCR. The resultant strain was transformed with the pDR244 vector containing the Cre recombinase under a temperature-sensitive promoter and grown at 37 °C without antibiotics to remove both the *erm*^*R*^ and *kan*^*R*^ cassettes – this generated the double knockout *ΔscmPyhaA* strain sensitive to antibiotics. The *ΔscmPyhaA* strain was transformed with BKE29290 gDNA and recovered in media containing 1 μg mL^-1^ erythromycin and 25 μg⋅mL^-1^ lincomycin. Successful recombination of *ytnL* with the *erm*^*R*^ cassette in the genome was verified *via* colony PCR, and the resistance cassette was removed as described above using pDR244 to generate a *ΔscmPyhaAytnL* strain. All deletions in the genome were verified *via* Sanger sequencing. Strains are listed in Supplementary Table 2.

### Production and purification of proteins

Proteins were expressed as described previously (48, 49). Briefly, *E*. *coli* strain BL21-(DE3)-RIPL was transformed with pET28 cloned with the coding sequences of *B*. *subtilis scmP*, *yhaA*, *ytnL*, and *ykuR* and recovered on LB containing 50 μg⋅mL^-1^ kanamycin. From single colonies, liquid cultures were grown at 37 °C in LB containing 50 μg⋅mL^-1^ kanamycin until the optical density at 600 nm reached 0.6. After cultures were cooled to room temperature, isopropyl β-d-thiogalactoside was added to 0.5 mM, and ethanol was added to 4% (v/v). Cultures were grown overnight at room temperature, and cells were harvested by centrifugation (4200*g*, 10 min).

Cell pellets were resuspended in 5 ml lysis buffer (50 mM Tris-HCl pH 8.0, 300 mM NaCl, 10 mM imidazole) and lysed by sonication using a Braun-Sonic 2000 set to 50% power for six, 15 s pulses, cooling on ice for 60 s between pulses. Cellular debris was removed from lysates *via* centrifugation (28000*g*, 10 min), and the supernatant was loaded onto a column containing 0.4 ml HisPur Ni-NTA resin (Thermo-Fisher Scientific) and washed with 9 ml wash buffer (50 mM Tris-HCl pH 8.0, 300 mM NaCl, 20 mM imidazole). Proteins were eluted with 0.5 ml elution buffer (50 mM Tris-HCl pH 8.0, 300 mM NaCl, 200 mM imidazole). Proteins were concentrated and desalted in an Amicon Ultra-4 10k MWCO spin column (Millipore) with desalting solution (100 mM KCl, 50 mM Tris-HCl pH 8.0). Glycerol was added to 10% (v/v) and 10 μl aliquots were snap-frozen in liquid nitrogen and stored at −80 °C.

### HPLC-based qualitative activity assays

To initially test whether recombinant ScmP, YhaA, YtnL, and YkuR possessed NAC deacetylase activity, endpoint assays were used. Assays (50 μl) contained 50 mM potassium phosphate (pH 7.8), 5 mM MgCl_2_, 4 mM NAC, and 1 μg of purified ScmP, YhaA, YtnL, or YkuR. Reactions were incubated for 10 min at 37 °C and stopped with the addition of 2 μl 1M HCl. 25 μl of each reaction were analyzed *via* RP-HPLC (Agilent 1100 series) with a Hypersil GOLD C18 column (250 x 4.6 mm) (ThermoFisher Scientific) and a mobile phase of 0.1% TFA, 3% acetonitrile running at 1 ml min^-1^, and detection at 200 nm.

### HPLC-based assays for metal affinity, substrate affinity, and kinetics

To determine metal affinity, assays (50 μl) contained 50 mM potassium phosphate (pH 7.8), 2 mM NAC, 5 mM metal salt (CoCl_2_, MnCl_2_, MgCl_2_, ZnCl_2_, NiCl_2_) or 10 mM EDTA, and 0.1 μg of purified ScmP, YhaA, or YtnL. Reactions were incubated for 4 min at 37 °C and stopped with the addition of 2 μl 1M HCl. 25 μl of each reaction were analyzed *via* RP-HPLC (Agilent 1100 series) with a Hypersil GOLD C18 column (250 x 4.6 mm) (ThermoFisher Scientific) and a mobile phase of 0.1% TFA, 3% acetonitrile running at 1 ml min^-1^, and detection at 200 nm. Each enzyme was tested with each metal in three independent assays.

To determine ScmP substrate affinity, assays (50 μl) consisted of 50 mM potassium phosphate (pH 7.8), 2 mM CoCl_2_, and 5 mM test substrate (*N*-acetylcysteine, N-acetylserine, *N*-acetylmethionine, *N*-acetylglutamic acid, *N*-acetylglycine, *N*_⍺_-acetyllysine, or *O*-acetylserine). Assays were started with the addition of either 1 μg ScmP or mock control and incubated for 15 min at 37 °C, then stopped with the addition of 2 μl 1M HCl. 10 μl of each reaction was analyzed *via via* RP-HPLC (Agilent 1100 series) with a Hypersil GOLD C18 column (250 x 4.6 mm) (ThermoFisher Scientific) with 0.1% TFA and 3% acetonitrile as the mobile phase, with detection at 200 nm. Substrates with detectable activity were tested with ScmP in three independent assays.

To determine kinetic parameters, assays (50 μl) contained 50 mM potassium phosphate (pH 7.8), 5 mM CoCl_2_, varying concentrations of NAC, and 0.1 μg of purified ScmP, YhaA, or YtnL. Reactions were incubated for 4 min at 37 °C and stopped with the addition of 2 μl 1M HCl. 25 μl of each reaction were analyzed *via via* RP-HPLC (Agilent 1100 series) with a Hypersil GOLD C18 column (250 x 4.6 mm) (ThermoFisher Scientific) and a mobile phase of 0.1% TFA, 3% acetonitrile running at 1 ml min^-1^, and detection at 200 nm. The amounts of NAC consumed and cysteine formed were determined by integrating diagnostic peaks using OpenLab software (Agilent) and comparing against standard curves. Kinetic parameters were calculated by fitting data to the Michaelis–Menten equation using GraphPad Prism software version 5.01. Each enzyme was tested at each concentration of NAC in three independent assays.

### LC-MS-based assays to determine product formation

To verify substrates and products of HPLC-based substrate tests, assays were analyzed *via* liquid chromatography-mass spectrometry (LC-MS). Assays (50 μl) consisted of 50 mM potassium phosphate (pH 7.8), 2 mM CoCl_2_, and 5 mM test substrate (*N*-acetylcysteine, *N*-acetylserine, *N*-acetylmethionine, *N*-acetylglutamic acid, *N*-acetylglycine, *N*_⍺_-acetyllysine, or *O*-acetylserine. Assays were started with the addition of either 1 μg ScmP or mock control and incubated for 15 min at 37 °C, then stopped with the addition of 2 μl 1M HCl and centrifuged (28000*g*, 5 min) through an Amicon Ultra-4 10k MWCO spin column (Millipore) to remove enzyme. Samples were diluted 1:10 in water, and 2 μl of each reaction was analyzed *via* LC-MS (Agilent 1260 Infinity II series) using an SB-C18 column (2.1 x 50 mm) (Agilent) with 0.1% formic acid as the mobile phase (0.4 ml min^-1^), 3000 V capillary voltage, and electrospray ionization with detection in positive mode. Diagnostic [M + H] ions for NAC and cysteine were validated with standards and are listed in Supplementary Table 3.

### Complementation growth assays

The pHCMC04 vector harboring the coding sequences for *scmP*, *yhaA*, or *ytnL* was transformed into the triple knockout *B*. *subtilis* strain generated in this study (*ΔscmPyhaAytnL*). As a control, the pHCMC04 empty vector (EV) was transformed into the following strains: 168 (WT), *ΔscmP*:*erm*^*R*^, *ΔyhaA*:*kan*^*R*^, *ΔytnL*:*erm*^*R*^, *ΔscmPyhaA*, and *ΔscmPyhaAytnL*. Positive transformants were grown overnight at 37°C in ED medium lacking sulfur (8 mM K_2_HPO_4_, 4.4 mM KH_2_PO_4_, 30 mM NH_4_Cl, 2.6 mM MgCl_2_, 0.3 mM Na_3_-citrate, 0.25 mM L-tryptophan, 0.1 mM FeCl_3_, 50 μM CaCl_2_, 5 μM MnCl_2_, 12 μM ZnCl_2_, 2.5 μM CuCl_2_, 2.5 μM CoCl_2_, 2.5 μM Na_2_MoO_4_, and 25 mM glucose) supplemented with 2 mM Na_2_SO_4_, washed twice in ED media lacking sulfur, and serially diluted to optical densities of 1.0, 0.2, 0.04, and 0.008. Five microliters of each dilution was spotted onto ED minimal medium plates with 1% (w/v) low-melt agarose, 0.2% (w/v) xylose, and 2 mM Na_2_SO_4_, 0.25 mM NAC, 0.25 mM 2SC, or no sulfur. Plates were incubated at 37 °C for 48 h and imaged. For liquid culture experiments, independent positive transformants were grown overnight at 37 °C in ED medium lacking sulfur supplemented with 2 mM Na_2_SO_4_, washed twice in ED media lacking sulfur, and serially diluted to an optical density of 1.0. Aliquots (15 μl) of washed cells were added to randomly assigned wells of a 48-well plate (Agilent) containing 285 μl of ED medium lacking sulfur supplemented with 20 mM NAC. Cultures were incubated in a BioTek Epoch 2 Microplate Spectrophotometer, at 37 °C for 24 h with continuous shaking, and OD_600_ measurements were taken every 30 min.

## Data availability

All data is either contained within the manuscript or will be shared upon request by contacting Thomas Niehaus (tniehaus@umn.edu).

## Supporting information

This article contains supporting information.

## Conflict of interest

The authors declare that they have no conflicts of interest with the contents of this article.
