# Supplementary material for: Bacillus subtilis encodes three N-acetylcysteine deacetylase enzymes that can catalyze the final step in S-(2-succino)cysteine breakdown
Source: J Biol Chem. 2025 Nov 17;302(1):110954. doi: 10.1016/j.jbc.2025.110954 (PMC12757645; doi:10.1016/j.jbc.2025.110954)
Supplement: Supporting Material [file mmc1.pdf]

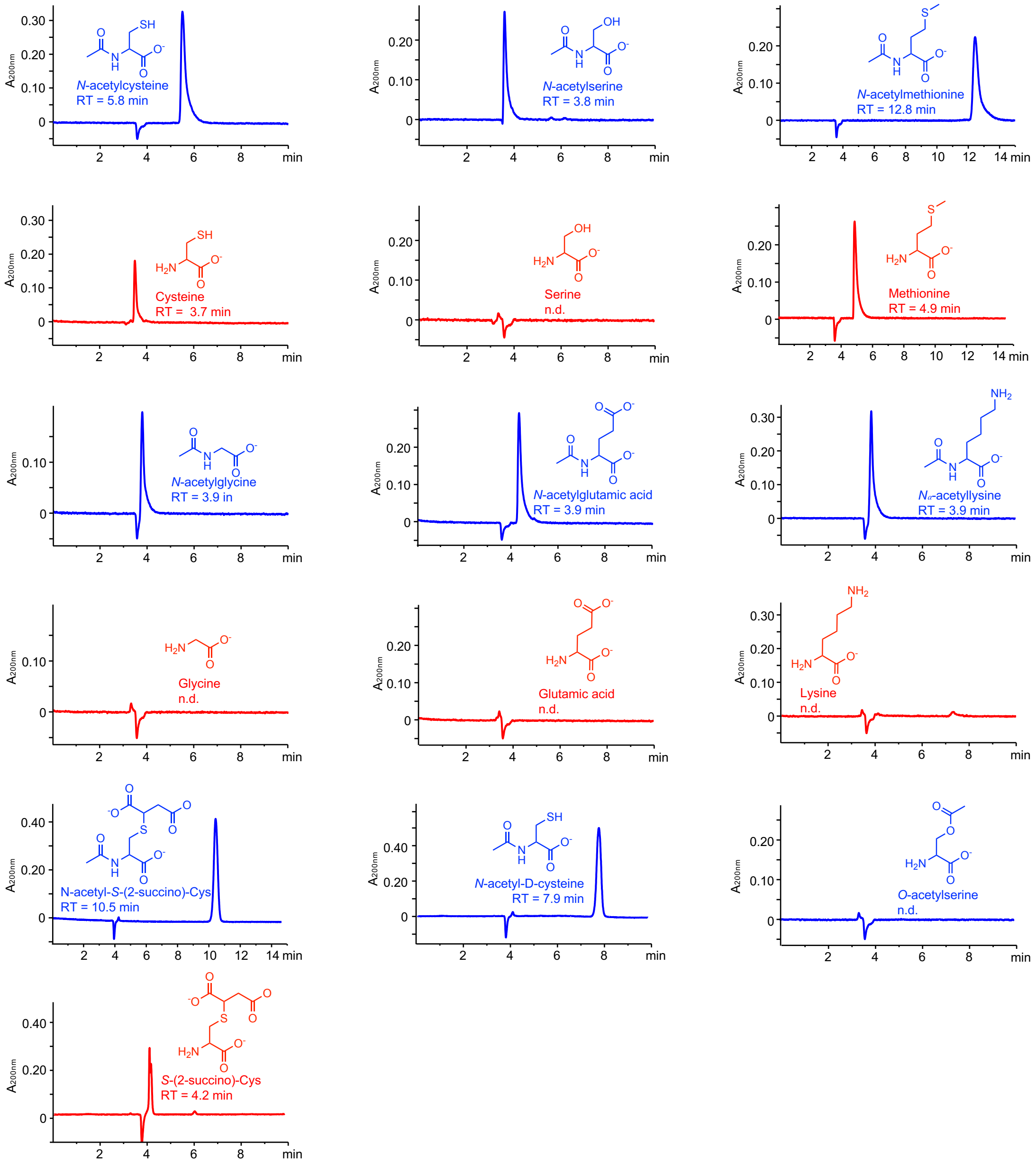

Supplemental Figure 1: Reference HPLC chromatograms for various amino acids and their acetyl- derivatives. For each compound 500 nmol was analyzed via HPLC on an Agilent 1100 series using a Hypersil GOLD™ 250 x 4.6 mm C18 column (ThermoFisher Scientific) with 0.1% TFA and 3% MeCN as the mobile phase, with detection at 200 nm. Glutamic acid, glycine, lysine, *O*-acetylserine, and serine were undetectable via these methods, while all other compounds generated strong, single peaks.

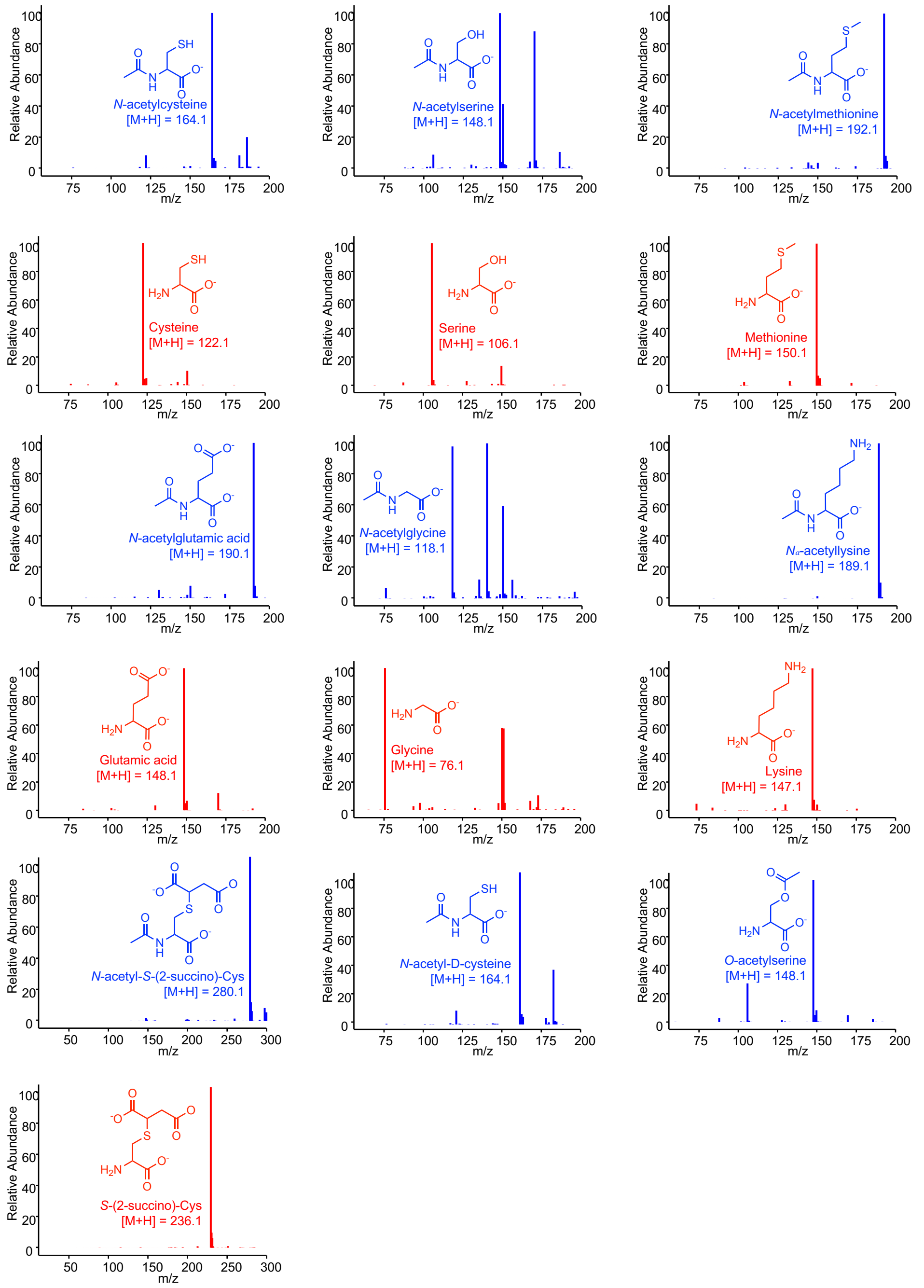

Supplemental Figure 2: Reference mass spectra for various amino acids and their acetyl-derivatives. For each compound, 1 nmol was analyzed via LCMS on an Agilent 1260 Infinity II series using an Agilent SB-C18 column (2.1x50 mm) with 0.1% formic acid as the mobile phase (0.4 $mL \cdot min^{-1}$), 3000 V capillary voltage, and electrospray ionization with detection in positive mode. All spectra were generated from the apex of the largest ion peak during the run. Blue spectra indicate acetylated amino acids, and red spectra are nonacetylated amino acids. All compounds yielded strong [M+H] peaks that were used as diagnostic masses in enzyme assays.

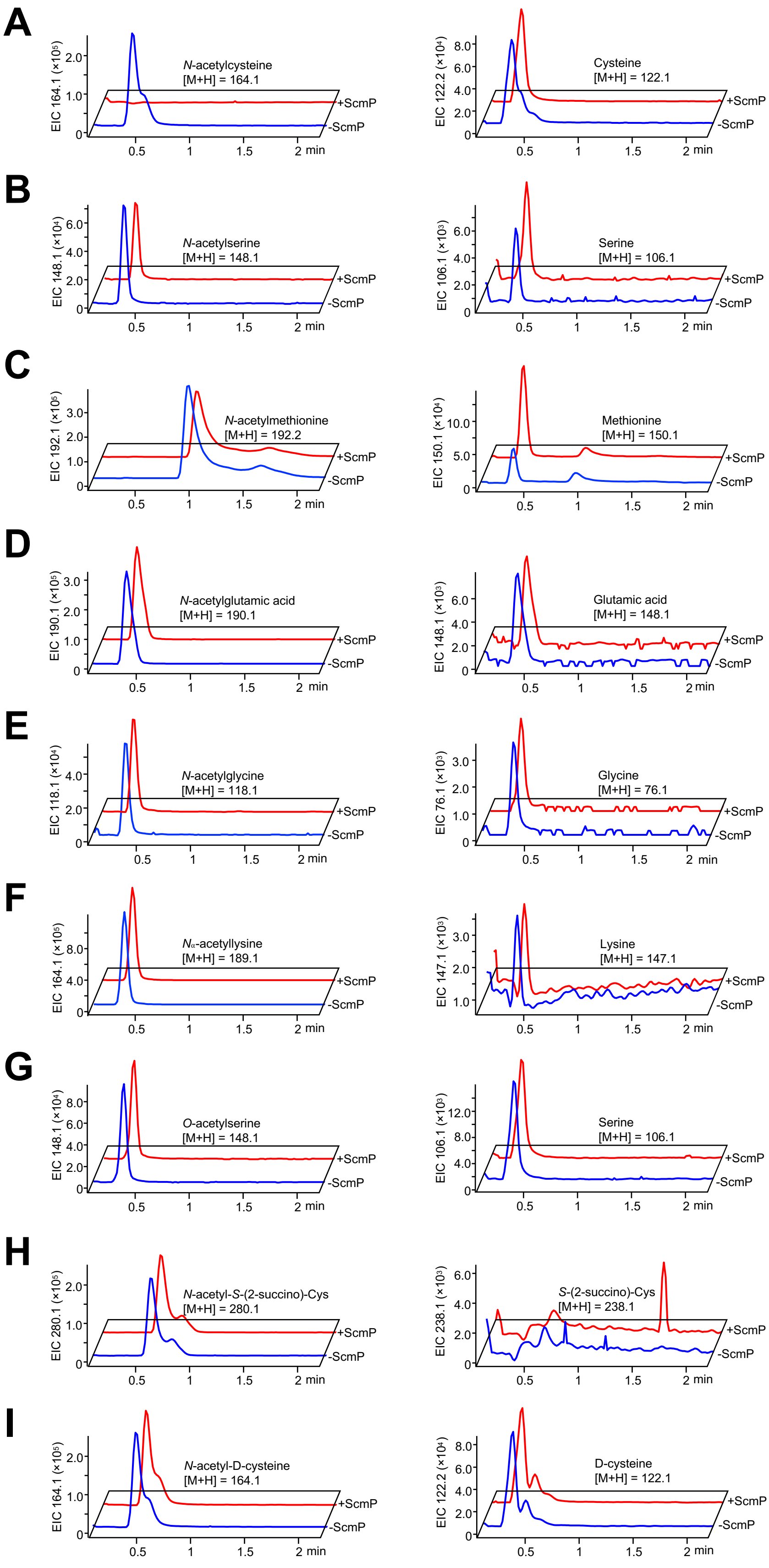

Supplemental Figure 3: ScmP is specific to *N*-acetylcysteine. Assays (50 µL) consisted of 50 mM potassium phosphate (pH 7.8), 2 mM $CoCl_2$, and 5 mM *N*-acetylcysteine (**A**), *N*-acetylserine (**B**), *N*-acetylmethionine (**C**), *N*-acetylglutamic acid (**D**), *N*-acetylglycine (**E**), N_α-acetyllysine (**F**), or *O*-acetyl-Ser (**G**). Assays were started with the addition of either 1 µg ScmP (red traces) or mock control (blue traces) and incubated for 15 minutes at 37°C, then stopped with the addition of 2 µL 1M HCl and centrifuged (28000g, 5 min) through a 20 kD filter to remove enzyme. Samples were diluted 1:10 in water, and 2 µL of each reaction was analyzed via LCMS on an Agilent 1260 Infinity II series using an Agilent SB-C18 column (2.1x50 mm) with 0.1% formic acid as the mobile phase (0.4 mL·min^{-1}), 3000 V capillary voltage, and electrospray ionization with detection in positive mode. [M+H] ion chromatograms were extracted from each sample for acetylated substrates (left column) and deacetylated products (right column).

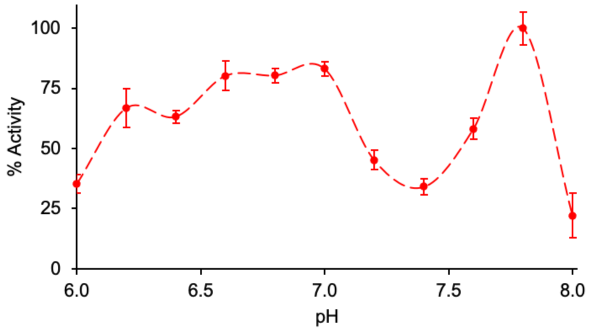

Supplementary Figure 4: ScmP activity is highest at pH 7.8. Assays (50 µL) consisted of 50 mM potassium phosphate (pH 6.0, 6.2, 6.4, 6.6, 6.8, 7.0, 7.2, 7.4, 7.6, 7.8, or 8.0), 2 mM $CoCl_2$, and 5 mM *N*-acetylcysteine. Assays were started with the addition of 0.1 µg ScmP and incubated for 4 minutes at 37°C, then stopped with the addition of 2 µL 1M HCl. 10 µL of each reaction was analyzed via HPLC on an Agilent 1100 series using a Hypersil GOLD™ 250 x 4.6 mm C18 column (ThermoFisher Scientific) with 0.1% TFA and 3% MeCN as the mobile phase, with detection at 200 nm. Bars represent SEM of 3 independent replicates

Supplementary Table 1: Oligonucleotide primers used in this study

Primer Name	Sequence
	Protein expression
Bs_scmP_NheI_F	ctagctagcatggccgacaaagcgtttcatac
Bs_scmP_XhoI_R	ttccgctcgagtcatttgatggtctccaatacaataaccg
Bs_yhaA_NheI_F	actggctagcttgtccatatccacactgcag
Bs_yhaA_HindIII_R	actgaagcttttatagctgatgataggtgatcgca
Bs_ytnL_NheI_F	actggctagcatgtctttggattattggagaaata
Bs_ytnL_XhoI_R	actgctcgagtcaatcagatatttttgactcttca
Bs_ykuR_NheI_F	actggctagcatgaagaaagaggagctcatc
Bs_ykuR_HindIII_R	actgaagcttttaatggcataaacagaaaaataacct
	Complementation assays
Bs_scmP_SpeI_F	atgcactagtatggccgacaaagcgtttcatac
Bs_scmP_BamHI_R	agctggatcctcatttgatggtctccaatacaataaccgc
Bs_yhaA_SpeI_F	atgcactagtttgtccatatccacactgcagaaagag
Bs_yhaA_BamHI_R	agctggatccttatagctgatgataggtgatcgcagc
Bs_ytnL_SpeI_F	atgcactagtatgtctttggattattggagaaatatagaaggc
Bs_ytnL_XmaI_R	atcgcccgggtcaatcagatatttttgactcttcattcgaatc
	Genotyping
Bs_scmP_up200	ggcgtttgcaaaagaggttgc
Bs_scmP_down200	gataacaggaacaaggggccgg
Bs_yhaA_up200	tcccatgggctttgattcccg
Bs_yhaA_down200	gactggagagtgttccagcagg
Bs_ytnL_up200	cggaattacacaagtagcttacaggcg
Bs_ytnL_down200	cactcccgatgcggcagtc
ErmR815	ccttaaaacatgcaggaattgacg
KanR774	agtaagtggctttattgatcttggg

Supplementary Table 2: Bacterial strains used in this study

Strain Name	Genotype/use	Source
	Bacillus subtilis	
168	WT	Koo *et al.* (2018)
BKE39740	*ΔscmP:erm*^{R}	Koo *et al.* (2018)
BKK10070	*ΔyhaA:kan*^{R}	Koo *et al.* (2018)
BKE29290	*ΔytnL:erm*^{R}	Koo *et al.* (2018)
	ΔscmPyhaA	This study
	ΔscmPyhaAytnL	This study
	Escherichia coli	
DH5α	Molecular cloning/preparation of vectors	Thermo-Fisher
BL21-(DE3)-RIPL	Recombinant protein expression	Agilent

Supplementary Table 3: Diagnostic masses used in LC-MS detection of deacetylase reactions

Compound	Ion	Mass
N-acetylcysteine	M+H	164.1
Cysteine	M+H	122.1
N-acetylserine	M+H	148.1
Serine	M+H	106.1
N-acetylmethionine	M+H	192.2
Methionine	M+H	150.1
N-acetylglutamic acid	M+H	190.1
Glutamic acid	M+H	148.1
N-actylglycine	M+H	118.1
Glycine	M+H	76.1
N-acetyllysine	M+H	189.1
Lysine	M+H	147.1
O-acetylserine	M+H	148.1
Serine	M+H	106.1
N-acetyl-*S*-(2-succino)cysteine	M+H	280.1
S-(2-succino)cysteine	M+H	238.1
N-acetyl-D-cysteine	M+H	164.1
D-cysteine	M+H	122.1
